# Fungal biotechnology: From yesterday to tomorrow

**DOI:** 10.3389/ffunb.2023.1135263

**Published:** 2023-03-27

**Authors:** Mitchell G. Roth, Nathaniel M. Westrick, Thomas T. Baldwin

**Affiliations:** ^1^ Department of Plant Pathology, The Ohio State University, Wooster, OH, United States; ^2^ Department of Plant Pathology, University of Wisconsin-Madison, Madison, WI, United States; ^3^ Department of Plant Pathology, North Dakota State University, Fargo, ND, United States

**Keywords:** fungi, biotechnology, technology, genes, sustainability, remediation

## Abstract

Fungi have been used to better the lives of everyday people and unravel the mysteries of higher eukaryotic organisms for decades. However, comparing progress and development stemming from fungal research to that of human, plant, and bacterial research, fungi remain largely understudied and underutilized. Recent commercial ventures have begun to gain popularity in society, providing a new surge of interest in fungi, mycelia, and potential new applications of these organisms to various aspects of research. Biotechnological advancements in fungal research cannot occur without intensive amounts of time, investments, and research tool development. In this review, we highlight past breakthroughs in fungal biotechnology, discuss requirements to advance fungal biotechnology even further, and touch on the horizon of new breakthroughs with the highest potential to positively impact both research and society.

## 1 Introduction

**Figure 1:**
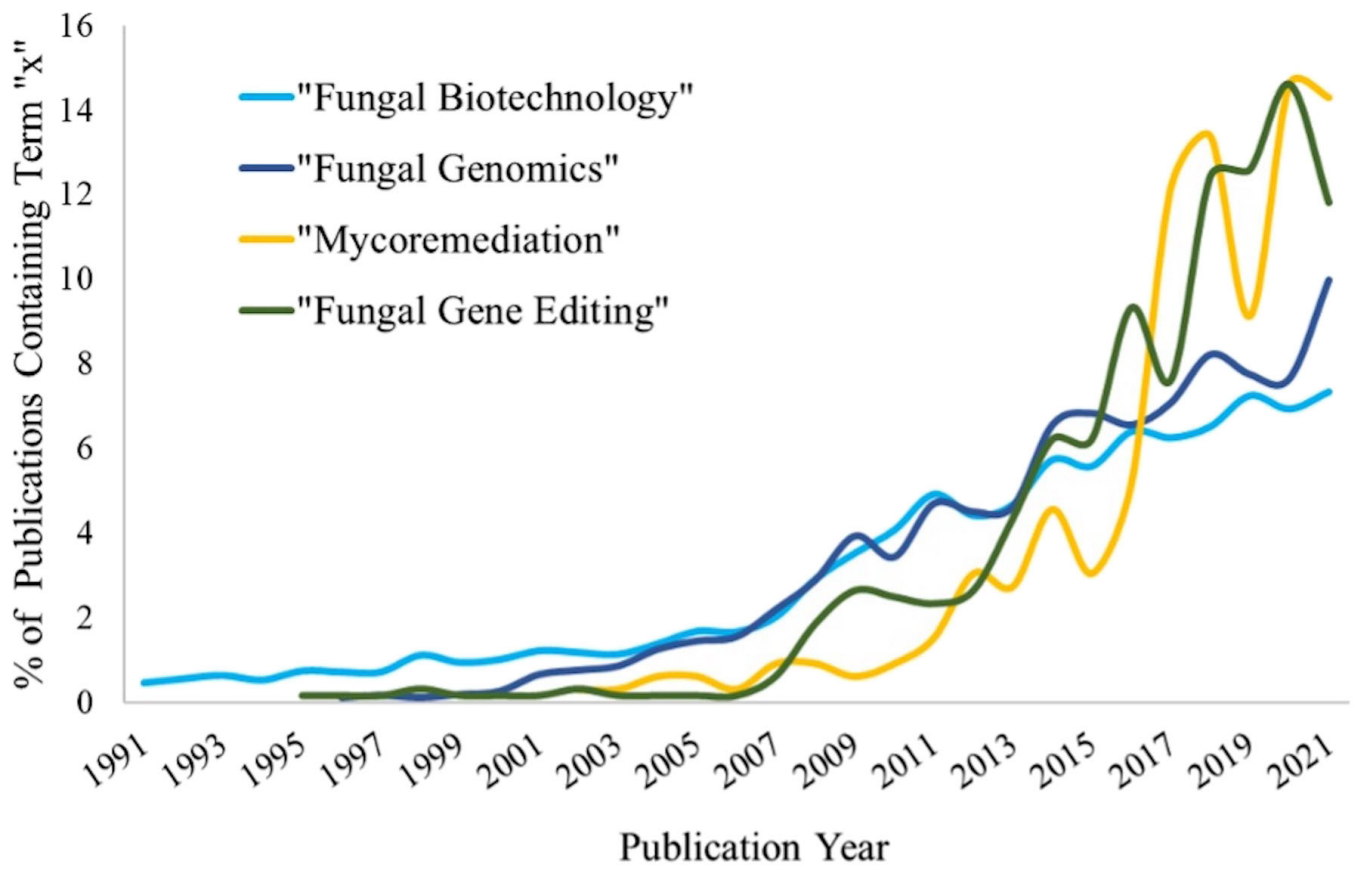
Representation of publication trends for given terms between 1991-2021. Publication numbers represent all documents found within the given years by Web of Science (www.webofscience.com/).

The fungal kingdom is vast and contains many different species with a variety of properties and useful enzymes. Fungi have contributed to the betterment of humanity throughout history and maintain a great deal of potential to further advance humanity. Fungi have been used for centuries in the food and beverage industry, used to make bread, cheese, beer, wine, and many other products (Hesseltine, 1965; Dupont et al., 2017). Since the 1990s, there has been a growing interest in using fungi for additional biotechnological purposes with a ten-fold increase in publications containing the terms associated with fungal biotechnology in recent years (**Figure 1**). Their genetic plasticity and ability to rapidly adapt to new hazardous and difficult to colonize environments means that fungi can contribute in many various environments. Their genetic tractability and transformability add to their overall plasticity, horizontally acquiring foreign genes, entire pathways, and even entire chromosomes. These properties make fungi ideal for industrial and pharmaceutical purposes, but also great subjects for studying genetic control of morphology and higher-level functions; to coordinate growth, sense their environment, respond to extreme conditions, and even mechanisms of basic cellular decision making. Additionally, fungi underpin and support our world’s entire ecological system. They reside in vast quantities in the soil beneath our feet, recycling nutrients and contributing to the food webs that many ecosystems rely on. Without their recycling of nutrients, the entire food web would cease to function. Yet, our understanding of fungi is more trivial than with nearly any other kingdom of life. This review aims to highlight their distinct advantages and where extra research would quicken the advent of breakthrough fungal technologies.

Broadly speaking, “biotechnology” is the industrial use of biological organisms and processes to benefit human endeavors. Fungal biotechnology is a specialized area of biotechnology that deals with the design and application of fungal biomass, metabolism, or genetics to address societal or environmental problems. The biology of fungi has great potential for addressing some of the world’s most pressing issues, such as food security, energy insecurity, human medicine, and environmental sustainability. Despite decades of research on fungi as decomposers, saprotrophs, and plant pathogens, applications of fungal biotechnology are only recently gaining significant traction. The future of fungal biotechnology looks very promising, with many new and exciting applications on the horizon.

Many fungi play a critical role in the environment by breaking down plant materials and recycling their nutrients and metabolic byproducts back into their environment. When yeasts, like *Saccharomyces cerevisiae* or *Yarrowia lipolytica*, break down plant materials through fermentation, the valuable product ethanol is produced (Bothast and Schlicher, 2005; Goldemberg, 2007; Liao et al., 2016; Adrio, 2017). Currently, plant-derived ethanol is mostly processed from yeast fermentation of simple sugars derived from milled corn starch and sugarcane. However, ethanol production can be more sustainable if produced from solely starch. Starting in the 1980’s, many studies made advancements in yeast engineering to improve ethanol production through the expression of other fungal amylases (Cole et al., 1988; Inlow et al., 1988; Ashikari et al., 1989; de Moraes et al., 1995). Yet, direct fermentation of starch remained elusive until additional engineering efforts to modify the yeast cell surface (Murai et al., 1997; Murai et al., 1999; Kondo et al., 2002; Shigechi et al., 2004a) and successful fermentation directly from starch is now possible (Shigechi et al., 2004b). These engineered yeast strains are equipped with glucoamylase gene from another fungus, *Rhizopus oryzae*, and an α-amylase gene from the bacterium *Streptococcus bovis* (Shigechi et al., 2004b).

Researchers have also set out to engineer *Saccharomyces cerevisiae* cells for production of fatty acid-derived biofuels and chemicals. To do this, they first deleted the gene encoding acetyl-CoA carboxylase (ACC1), which is responsible for converting acetyl-CoA to malonyl-CoA. The researchers then inserted a gene encoding thioesterase (TE) into the ACC1 locus. TE is an enzyme that catalyzes the hydrolysis of acyl-CoAs to free fatty acids. The engineered *S. cerevisiae* cells were able to grow on glucose and produce high levels of free fatty acids, which can be used for biofuel or chemical production (Hu et al., 2019). Further research on fungal-derived biofuel production is critical to an era of social and legislative efforts to reduce dependence on a depleting resource of fossil fuels. Further research is being done to investigate other ways these fungal-derived biodiesel, biofuels, and ethanol can be utilized. Since ethanol production requires the presence of plant materials and fungal materials, the sustainability of biofuel production holds significant promise. The world’s dependence on fossil fuels despite its ever-declining availability means there is a growing need for renewable sources of energy like biofuels. Fungal-derived biofuels offer a more sustainable and environmentally friendly alternative to traditional fossil fuels. As a model organism with the first genome to be fully sequenced, *S. cerevisiae* is being further explored as a tool to accomplish other complex biochemical processes, including engineering of nitrogen-fixation potential to address issues of nitrogen fertilizer sustainability (Burén et al., 2017).

Plants are not the only materials that fungi have been successful at breaking down. Some fungi have unique abilities to break down hazardous hydrocarbons, organic pollutants, and other toxic phenolic compounds (Treu and Falandysz, 2017). Fungi have shown so much potential for this that the entire field has been dubbed ‘mycoremediation’. Nutrient pollution, specifically nitrogen and phosphorus, is one of the main environmental problems in natural waterways and both salt and freshwater ecosystems from agricultural fertilizer runoff. Fungal biomass from *Trichothecium roseum* can remove up to 97.5% of phosphate; other species such as *Epicoccum nigrum*, *Geotrichum candidum*, and other *Trichoderma* sp. can remove significant amounts of nitrogen from waste water streams (Coulibaly et al., 2003; Sankaran et al., 2010), removal of heavy metals (Shakya et al., 2016), waste from the textile industry (Jebapriya and Gnanadoss, 2013), and agro-industrial waste (Ferreira et al., 2016; Matei et al., 2021). Both filamentous (Al-Otibi et al., 2022) and edible mushroom species like *Pleurotus tuber-regium* (oyster mushroom) and *Fistulina hepatica* (beefsteak mushroom) (Isikhuemhen et al., 2003; Shen and Chaichi, 2020) have demonstrated the ability to bioremediate crude oil hydrocarbons. One successful mycoremediation experiment was the cleanup of an oil spill in Prince William Sound, Alaska. In 1989, the Exxon Valdez spilled over 11 million gallons of oil into the sound. The oil coated beaches and killed wildlife. A team of scientists used fungi to break down the oil by spraying a mixture of calcium carbonate and fungal spores onto the beaches, allowing the fungi to grow and break down the oil. This project demonstrated that mycoremediation can be an effective way to clean up large-scale environmental disasters (Kumar and Kaur, 2018).

When the rose-pink yeast *Rhodotorula taiwanensis* was first discovered in radioactive waste sites, researchers recognized the capacity for this fungus to mycoremediate these waste sites. Characterization of the genome of this robust yeast species revealed the prosperity for sulfur metabolism to break down sulfate-compounds present in acid mine drainage, genes involved in heavy metal resistance and acquisition (including uranium), high tolerance for radioactivity (Tkavc et al., 2018). In addition, *R. taiwanensis* can also reduce the bioavailability of the heavy metals and radionuclides, making them less mobile and less likely to contaminate groundwater or other ecosystems. *R. taiwanensis* has great potential as a tool for cleaning up radioactive waste sites, however, more work is needed to optimize this remediation process and the genes involved have yet to be fully explored and utilized.

Fungi are also poised to help address food security issues in many unique ways. Cultivation of fungi as food in the form of edible mushrooms dates back to as early as 200 BC in China, where special notes and documentation about the effects of environment on the growth and appearance of various *Auricularia* spp. (Cheng and Tu, 1978). Today, *Agaricus bisporus* button mushrooms are a staple in produce sections of grocery stores around the globe, including the United States. Other mushrooms like *Flammulina velutipes*, the enoki mushroom, are common in East Asian produce stores. Many other food favorites require fungal fermentation for their unique flavors, like tempeh produced by fermentation with *Rhizopus oryzae*, soy sauce produced through fermentation by *Aspergillus oryzae*, blue cheese colonized by *Penicillium roqueforti*, and salami aged and seasoned *via* colonization by unique *Penicillium* species, like the recently described *P. salamii* (Perrone et al., 2015). More recent efforts to utilize fungi or fungal-derived products in food has led to the development of unique products with meat-like properties (hamburger, bacon, etc. - Impossible burger, Quorn, and MyBacon), with little or no animal products involved (Meyer et al., 2020). Beyond fungi as food themselves, many fungi form intricate, beneficial relationships with plants, including food crops and trees, in the form of mycorrhizae. Mycorrhizal fungi that form intimate relationships with plant roots and exchange nutrients with them, which can help improve crop yields, create more nutritious foods, and even help crops withstand pests and diseases (Jeffries and Rhodes, 1987). With the world population projected to reach 9 billion by 2050, there is an urgent need to find ways to increase food production while ensuring food security for all people. Fungal biotechnology through new food products and enhancing mycorrhizal relationships offers promising solutions to this challenge (Thirkell et al., 2017).

**Figure 2:**
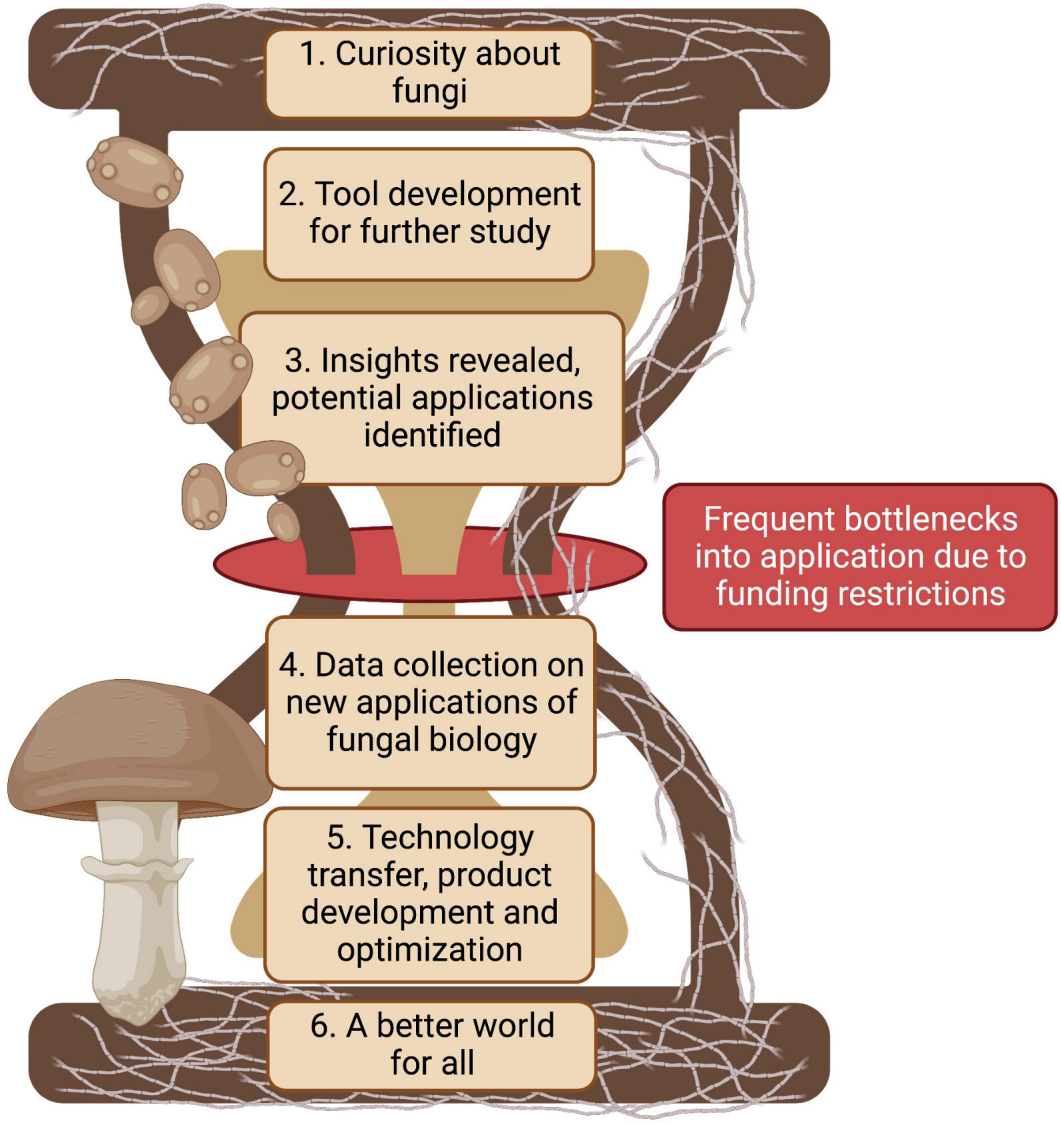
Fungi contribute to environmental cycles in many ways. Broad curiosity about fungi begs the necessity of tool development to better understand how they do the things they do. Better tools reveal deeper insights that can have novel applications in our daily world. Promising applications need further funding support for technology transfer, product development, and optimization in order to help contribute to a better world for all. Created with BioRender.com.

Advancements in biotechnology require intricate knowledge of the biological systems and creative thinking in potential applications. All of this requires financial investments in research. Fungal biotechnology is likely to advance by following phases highlighted in **Figure 2**: building a curiosity about fungi, developing tools for advancing our knowledge, generating new insights from studies, applying the new knowledge in unique ways, transferring, developing, and testing the new technologies, leading to a better world for all. Each successive phase is built upon the previous one to create a more complete understanding of these organisms and their potential applications in the real world. The first phase of fungal biotechnology is simply a curiosity about fungi to further study fungal species and attributes. Only a small portion of the overall diversity of fungi e.g. *Aspergillus*, *Neurospora*, *Saccharomyces*, *Fusarium*, etc. have made it to model organism status. These organisms were selected due to their ease as an experimental model or dire need to control as a pathogen. Understanding other fungi and the roles they play in a broader context will lead to the development of tools for studying fungi, such as advanced microscopes and culturing techniques for more difficult to culture fungi. None of this is possible without adequate funding for basic mycological research.

## 2 MycoGenetics: Applications of fungal genetics

The impact of next generation sequencing technology on biology has been profound, particularly in the world of fungal genetic research. Information revealed from improved DNA sequencing has reshaped the entire phylogeny of the fungal kingdom. In many cases, DNA sequencing has revealed that different fungi thought to be the same species were in fact very distinct from one another and vice versa (Begerow et al., 2010; SanMiguel, 2011; Bazzicalupo et al., 2013; Dannemiller et al., 2013; Ahrendt et al., 2018; Forin et al., 2018). Comparing DNA sequences of many fungi that were thought to be very different species instead revealed identical sequences and demonstrated the vast complexity of different fungal reproductive structures (Axelson-Fisk and Sunnerhagen, 2005; Schoch et al., 2014; Wu et al., 2019).Though this discovery has led to some confusion and controversy over which name to select for a given fungal species (i.e. “One Fungus, One Name”) (Taylor, 2011), the benefits of DNA sequencing in fungi far outweigh the negatives. Combining whole-genome sequencing with other developing ‘-omic technologies like phenomics, transcriptomics, and metabolomics has allowed researchers to understand fungi and their interactions with the environment like never before.

### 2.1 Advancements through long-read and other emerging sequencing technologies

Fungal genomes are notoriously difficult to sequence and assemble due to their large size and repetitive sequences. As a result, most fungal genome studies have relied on short read sequencing technologies, which often produce fragmented assemblies. However, recent advances in long read sequencing technologies, such as Nanopore and PacBio, have made it possible to generate high-quality fungal genome assemblies. These new technology platforms have already had a major impact on fungal genetic research. For example, they have been used to generate the first complete assembly of the human pathogenic fungus *Cryptococcus neoformans* (Passer et al., 2019). This fungus is responsible for hundreds of thousands of deaths each year, so having a high-quality genome assembly is a major breakthrough for understanding the genetic mechanisms of the fungus to incite disease in humans. The ability to generate complete genomes for this and other medically important fungi will help researchers develop new treatments and diagnostic tools.

In addition, long read sequencing has also been used to discover novel genes and pathways in fungi. For example, a recent study used PacBio long reads to annotate over 7,000 previously uncharacterized genes in the model fungus *Saccharomyces cerevisiae* (McIlwain et al., 2016). These newly discovered genes are involved in a variety of functions including metabolism, stress response, and cell wall biogenesis. The identification of these genes would not have been possible without long read sequencing. Long-read technologies are enabling researchers to generate high-quality genome assemblies for medically important fungi and discover novel genes and pathways that were previously hidden in fragmented short read data sets.

Another sequencing advancement, Hi-C sequencing, has contributed to our understanding of fungal genome structure. Hi-C sequencing provides detailed information about the three-dimensional (3D) structure of chromosomes and genomes. Hi-C data has been used to generate 3D models of several fungal genomes, including those of the yeast *Saccharomyces cerevisiae* and the human pathogen *Candida albicans* (Schalbetter et al., 2019; Guin et al., 2020). These models have revealed important insights into the organization and function of these genomes. For example, they have shown that *S. cerevisiae* chromosomes are organized into distinct compartments that contain different sets of genes involved in specific biological processes. Additionally, the 3D model of *C. albicans* showed that this pathogen has a highly dynamic genome that undergoes large-scale changes in structure during its infectious cycle. These studies demonstrate the power of Hi-C sequencing for investigating the 3D structure of fungal genomes. This technique is likely to be particularly useful for studying species with complex or unusual genome structures, such as those found in many plant pathogens.

In recent years, machine learning has become increasingly popular in many science fields, including mycology. Machine learning algorithms are able to effectively process large amounts of data and identify patterns that would be difficult for humans to discern. Additionally, machine learning can be used to develop predictive models that can be used to generate new hypotheses or guide experiments. There are a number of different machine learning algorithms that have been applied in fungal research, including support vector machines, decision trees, and artificial neural networks. For instance, these algorithms have helped predict virulence and fungicide tolerance in clinical isolates of many fungi (Chaudhury et al., 2011; Dix et al., 2015; Delavy et al., 2020). Additional research applying machine learning to fungal biology is certain to help further identify patterns in data sets and generate hypotheses, predictions, and new knowledge.

High quality whole-genome sequences of fungi provide a valuable resource for the fungal biotech community. As machine learning algorithms continue to improve predictions of gene identification and protein 3D structures, these resources will contribute to new alleles of known genes, entirely new genes, and whole gene clusters. Equipped with these resources, fungal geneticists can study fungal genes and their products for additional contributions to benefit society.

### 2.2 Bioinformatics tools for characterizing fungal proteins

In the past decade, research on fungal proteins has accelerated due to the availability of more sophisticated predictive tools. SignalP and EffectorP are two such tools that predict the secretion of proteins in fungi. SignalP consists of a neural network that is trained on a set of known signal peptides. It can be used to predict whether a given protein sequence contains a signal peptide that destines a protein for secretion (Almagro Armenteros et al., 2019). EffectorP consists of a Support Vector Machine that is trained on known effector proteins. It can be used to predict whether a given protein sequence is an effector protein.

ApoplastP is used to predict the localization of proteins in the plant apoplast, and Localizer is used to predict additional subcellular localizations in plant cells (Sperschneider et al., 2018). ApoplastP uses four different methods (sequence alignment, structural analysis, hydrophobicity analysis, and subcellular localization prediction) to predict the localization of proteins in the apoplast. Localizer uses sequence data and sliding windows to predict signals for chloroplast, mitochondrial, and nuclear localization signals (Sperschneider et al., 2017).

While subcellular localization prediction is often defined by amino acid chemistry and motifs, other features such as enzymatic activity and ligand binding site prediction are less amenable to such an approach. Difficulty in establishing the 3D structure of a protein from its sequence, or the “protein folding problem”, has been an ongoing issue in biology, especially for proteins with no experimentally validated homologs (Abriata et al., 2019; Pearce and Zhang, 2021). Despite billions of known protein sequences, the list of those with known structures is in the thousands and fungal proteins represent a bare fraction, leading to limited adoption of these tools in the fungal research community (Jumper et al., 2021). Recent advances in machine learning, however, have led to the development of the protein structure prediction tool AlphaFold, which allows for atomic level prediction of protein structure even in the absence of characterized homologs (Jumper et al., 2021). Since the release of AlphaFold, its potential for revolutionizing pharmaceutical treatment of human fungal pathogens has been discussed (Thornton et al., 2021). Additionally, AlphaFold has been used in the analysis of a broad range of fungal plant pathogens to uncover common structures of secreted virulence proteins which have lost sequence similarity across large evolutionary timescales (Seong and Krasileva, 2023). Although this tool will require extensive validation in the future to confirm its value in fungal protein modelling, the potential for using AlphaFold in rational protein design to achieve valuable and/or novel enzymatic activity in fungi is promising.

Fungal proteins are involved in a variety of important biological processes, including pathogenesis, metabolism, and cell-cycle regulation. The use of predictive tools has helped to accelerate research on fungal proteins by providing information about the function and localization of these proteins (Stergiopoulos and de Wit, 2009; Petre and Kamoun, 2014; Sperschneider et al., 2015). These tools will be useful in furthering our understanding of fungal proteins and their roles in disease and development of products involving fungal products derived from fungal proteins including enzymes, antibiotics, and vitamins. As more data becomes available, it is likely that machine learning will play an even bigger role in fungal research.

Gene annotation software helps reveal conserved regions of genes across organisms. Accurate prediction tools helps us understand how fungal genes in genomes are structured and organized, and how evolutionary principles may be affecting the same sets of genes in different fungal species. These tools are sure to help influence fungal biotechnology efforts, as key findings in the conservation of gene structures also helps us understand conserved function between fungal species.

### 2.3 Single-cell RNA sequencing of fungal cells

scRNA-Seq is a technology which builds on previous advancements in high-throughput sequencing by allowing for the targeted analysis of gene expression in individual cells. This is first achieved through the isolation of cells through methods including laser microdissection, flow cytometry, and manual cell picking, followed by the preparation of a cDNA library (Gross et al., 2015). This approach has already been transformative in our understanding of medicine (Tang et al., 2019), plant physiology (Rich-Griffin et al., 2020), and bacterial ecology (Mauger et al., 2022), but has seen limited application within fungal biology. Mycology often trails in technological advancements in these fields, missing out on exploring the unique aspects of both yeast and filamentous fungi on these platforms.

A logical application of this technology is in better understanding populations of yeast acting in either natural or manufacturing settings. Unlike multicellular organisms typically requiring harsh microdissection or protoplasting to release individual cells, yeast are inherently single-celled organisms that lend themselves to being more easily sorted and lysed. To this goal, scRNA-Seq has been utilized on *Saccharomyces cerevisiae*, *Candida albicans*, and the fission yeast *Schizosaccharomyces pombe* (Lipson et al., 2009; Gasch et al., 2017; Saint et al., 2019; Dohn et al., 2021), all of which demonstrate a surprising level of transcriptomic heterogeneity in seemingly homogenous populations. Multiple studies have demonstrated the existence of subpopulations within isogenic yeast cultures which respond distinctly to both stress-inducing and growth-promoting conditions, as has been noted in human and mouse models (Gasch et al., 2017; Tan and Wilkinson, 2020; Urbonaite et al., 2021). While scRNA-Seq has been primarily focused on answering questions of basic molecular and microbiology, a more applied use of this method might be in understanding yeast in an industrial setting (i.e. growth in bioreactors). Evaluating gene expression of yeast populations during biomass/lipid production has been a major research focus in recent years, and a more precise understanding of transcriptomic shifts in these populations may allow for a greater fine-tuning of targeted biochemistry (Aliyu et al., 2021). Multiple, easily scalable microfluidics approaches to yeast single-cell transcriptomics have been developed in recent years, suggesting that we may be at the forefront of this technology gaining wider application within basic and applied research (Dohn et al., 2021; Urbonaite et al., 2021).

Similar to yeast cells, filamentous fungi demonstrate heterogeneity across colonies and even across cells within a given hypha (de Bekker et al., 2011; Tegelaar and Wösten, 2017). Apical cells are responsible for hyphal extension and are responsible for a majority of protein secretion, but appear largely non-dependent on sub-apical cells during growth in culture (although this is not the case during other processes including pathogenesis) (Tegelaar and Wösten, 2017; Peyraud et al., 2019). It has been previously suggested that an increased focus on the molecular underpinnings of fungal apical cells over -omics analysis of whole colonies may help to reduce cellular heterogeneity and optimize the fungal production of enzymes and other chemicals (Wösten, 2019). scRNA-seq presents a distinct opportunity to address these concerns, as the transcriptomic activity of apical cells can avoid being masked by less productive cells within the same colony/batch. This approach comes with additional challenges, however, as there has been very little method development focused on scRNA-seq in filamentous fungi.

Efforts to build these tools could expedite fungal biotechnology to identify key genes involved in different fungal processes of the same fungal isolate. Both fungi and slime molds have demonstrated “decision-making” abilities (Beekman and Latty, 2015; Money, 2021), and understanding how cell-to-cell communication occurs and gene expression of individual cells at a hyphal tip compared to a mature mycelial mat are sure to reveal novel insights with biotechnological value. For instance, knowing what genes are important to initial colonization of a new environment compared to the genes needed to establish dormancy or initiate fruiting in that new environment could help improve efficiency of bioremediation efforts.

### 2.4 Polymutants in fungal research

For decades, the creation of “gene knockouts’’ has been the premier method used by the scientific community to study the role and importance of specific genes in fungal biochemistry, host interactions, and development. While the generation of single gene mutants has helped to elucidate some of the most critical processes in fungal physiology and biochemistry, the limitations of such mutants are apparent (Teng et al., 2013). Fungal genomes often have highly expanded gene repertoires, including cell wall degrading enzymes, effectors, hydrophobins, and laccases which may work individually or in concert to achieve defined and dynamic goals (Westrick et al., 2019) Given these expansions, there can be significant controversy surrounding the relative importance of a given gene in any biological process, especially in situations in which multiple genes may have tissue specific or coordinated functions (Arroyo-Velez et al., 2020). A solution to this controversy is the generation of polymutants, in which multiple members of a gene family or biosynthetic pathway have been removed from the genome.

An attractive approach to remediating this problem is the usage of marker-independent CRISPR-Cas9 technology, which is capable of acting in tandem with a small guide RNA (sgRNA) to induce small insertions or deletions within target genes and abolish protein production (Ouedraogo and Tsang, 2020). Although Cas9 was first used in yeast in 2012 (Qi et al., 2012) and a filamentous fungus in 2015 (Liu et al., 2015), an explosion of interest in recent years highlights the potential of this tool (Hahn and Scalliet, 2021). A case study of this technique can be seen in the necrotrophic fungal pathogen *Botrytis cinerea*, in which a 12x polymutant was generated using recombinant Cas9 to assess the relative importance of cell death inducing proteins during infection (Leisen et al., 2020; Leisen et al., 2022). Given the importance of cell death induction for such a pathogen, it may be unsurprising that most individual genes had a negligible effect on virulence, but two distinct polygolacturonases were confirmed to be critical. The ease of such transformation techniques has the additional benefit of allowing for the cross-validation of gene knockouts between research labs, which is important given the concern of reproducibility of individual gene knockout phenotypes (Leisen et al., 2022; Qin et al., 2023).

Despite the convenience of the CRISPR-Cas9 technique, some fungal systems must contend with the issues surrounding nuclear localization/cytotoxicity of Cas9 and/or poor production of small guide RNA (Arazoe et al., 2015; Fang et al., 2017; Ah-Fong et al., 2021). In these cases, an additional tool which has gained interest in recent years are recyclable markers, which allow for the sequential removal of genes using a single selection marker, which is subsequently excised from the genome between each transformation. This technique has been demonstrated in a range of fungal organisms using both antibiotic and auxotrophic markers (Khrunyk et al., 2010; Zhang et al., 2013; Garcia et al., 2017). Such a system was used to generate a septuple knockout mutant of all putative hydrophobin genes in the genome of *Penicillium expansum* (Luciano-Rosario et al., 2022).

While the benefits of these technologies to fundamental biology are clear, their potential relevance in fungal biotechnology cannot be overstated. Fungal organisms often undergo extensive gene editing to achieve desired characteristics for secondary metabolite (Ning et al., 2022), lipid (Coradetti et al., 2018), or biomass production (Wilson and Harrison, 2021). Polymutants will likely be transformative in our understanding of fungal biochemistry, as gene redundancy can often mask the importance of given proteins in a biological function (Dalmais et al., 2011). Although both of the techniques described here have existed for close to a decade, increasing adoption in recent years suggest that polymutants will likely become a gold standard of fungal research. On the opposite end of the spectrum to polymutants, transgene addition to fungal strains and culturing in bioreactors can also be valuable. For example, yeast strains have been engineered as cellular factories for insulin production (Baeshen et al., 2014). More examples of medicinal and agricultural applications of fungal metabolism and bioreactors are described in section 3.2.

## 3 MycoImplication: The applications of knowledge derived from research on fungi

### 3.1 Sustainable biomaterials

A primary component of all fungal cell walls is chitin. Like cellulose, chitin can be processed into many different end-use products with broad-reaching applications (Meyer et al., 2020). With the ability to thrive on waste and by-products of current industries that utilize plant material, fungi can be exploited as cellular factories to produce chitin, chitosan, and other desirable end-products.

The ability for fungal mycelia to colonize substrates in a filamentous, interwoven manner provides unique opportunities to make biomaterials in specific shapes and sizes. This combination of traits makes fungi an appealing source of sustainable biomaterials. When mycelia are inoculated onto plant material, it takes the shape of whatever container that houses the plant substrate while consuming the nutrients from the plant matter. When dried, the mycelial product can be strong, durable, lightweight, thermotolerant, and flame resistant (Jones et al., 2020; Mojumdar et al., 2021). These materials have begun making their way into society in the form of packing and shipping materials, which are attractive to customers craving enhanced sustainability. Mycelial biomass is also amenable to 3D printing processes, allowing customizable shapes of mycelial composite materials (Bhardwaj et al., 2020).

Investigations into the compression strength of mycelial bricks give promise for a future where foundations of buildings and other structures could be supplemented with sustainable alternatives to concrete (Achal and Mukherjee, 2015; Ziegler et al., 2016; Jones et al., 2020; Ghazvinian and Gürsoy, 2022). Concrete frequently heaves and cracks in colder climates due to the freezing and thawing of the seasons. Some Ascomycete fungi metabolically induce the precipitation of calcite, an important component of limestone and concrete, leading to the notion of ‘self-healing concrete’ (Li et al., 2015; Khushnood et al., 2022). Further, the hydrophobic nature of most mycelia could help improve the longevity of concrete structures by helping it shed water (Khushnood et al., 2022). Additional creative, forward-thinking uses of mycelium are likely to be proposed and pursued as interests in mycology and sustainability continue to grow.

### 3.2 Bioreactors

As such a diverse kingdom, fungi produce and accumulate a diverse range of unique compounds. These compounds are being studied in many disciplines for their potential applications. Many consumers enjoy the fungal-derived fermentation products like bread, tempeh, kombucha, wine, or beer, and active efforts are underway to improve fermentation processes for precision, quality, and safety in fermented foods (Chai et al., 2022). Milk spoiled “the right way” can produce delicious cheese, but requires the addition of chymosin and pepsin that was historically harvested from calf stomachs. Today, these proteins are produced by *Aspergillus niger* in mass quantities in bioreactors (Dunn-Coleman et al., 1991). Fungi produce many other diverse ingredients and additives that are critical for our food systems, including an estimated 95% of the citric acid used in the food industry produced by *Aspergillus niger* (Copetti, 2019). Beyond traditional fermentation, yeasts and other fungi have begun to receive renewed attention for their roles in bioreactors for mass-scale production of other desired compounds.

Historically, research on fungal bioreactors was intended to identify ways to produce large quantities of plant degrading enzymes, such as cellulases, pectinases, xylanases, and other ligninolytic enzymes (Cocking, 1972). Today, the field of “white biotechnology” refers to the use of biological organisms to mass produce these compounds and more, with renewed focus on fungal contributions (Meena and Siddhardha, 2019). Many fungal species across the globe fill a similar niche in their contributions to the degradation of plant products. Therefore, many alleles of these catabolic enzymes exist in nature, and efforts to identify fungal strains with highly inducible production of these enzymes in submerged fermentation vessels have been successfully identified (Fazenda et al., 2008; Hansen et al., 2015). Additional fungal-derived enzymes like hydrolases, lipases, amylases, and proteases contribute to more efficient detergents that we use in daily activities like laundry and dishwashing (Østergaard and Olsen, 2011).

Similarly, the field of “red biotechnology” refers to the use of biological organisms to produce medical tools and medicinal compounds. Fungal metabolites long been recognized for their potential to produce unique secondary metabolites. When purified, these compounds often have characteristics of pharmaceutical or agricultural relevance. Key examples of fungal compounds with medicinal application include the spurious discovery of penicillin in 1928 by Alexander Fleming in *Penicillium rubens*, cyclosporine produced by *Tolypocladium inflatum*, statins produced by *Aspergillus* and *Penicillium* species, and stereochemistry transformation of steroidal hormones by certain *Rhizopus* species (Aly et al., 2011). Further, the anti-cancer drug taxol originally discovered in 1962 from the Yew tree, *Taxus brevifolia*, can also be produced in Yew-tree associated mycorrhizal fungi, *Taxomyces andreanae* (Stierle et al., 1993). Further research has discovered that other mycorrizhal fungi produce taxol, and advancements in fungal biotechnology are likely to improve production of this important medicinal compound (Gond et al., 2014). The strobilurins are another class of metabolite with key applications in agriculture. Ironically, the strobilurin fungicides that successfully kill many fungal pathogens of plants were discovered in the Basidiomycete *Strobilurus tenacellus* (Feng et al., 2020).

In the current era of mass genome sequencing efforts, new biosynthetic pathways are being discovered in non-model fungi, leading to new avenues for red biotechnology and fungal contributions to medicine and beyond (Erjavec et al., 2012; Wilken et al., 2019). A broadened survey of more fungal species for metabolite production is key to identifying new compounds with medicinal or agricultural relevance.

### 3.3 Fungal batteries

The future of fungal applications is electric! Though passionate mycologists are likely to agree figuratively, this can also be taken quite literally. The porous structure of mushroom flesh can be processed into carbon-rich, porous nano-ribbons, providing unique qualities and abilities to hold and release electrical currents, providing great potential to improve ion flow in Lithium-sulfur batteries (Campbell et al., 2015; Wu et al., 2016). Further, the classic “toadstool” mushroom, *Amanita muscaria*, and others in the *Amanita* genus frequently produce a compound called Amavadin, which has a vanadium ion in its core. Vanadium is relatively rare in nature, but has tremendous potential to contribute to the next generation of battery production *via* redox flow batteries (Kim et al., 2015; Egitto et al., 2022). Investigating ways to promote vanadium accumulation in mushrooms provides a promising avenue for producing more efficient and sustainable batteries in a world that is becoming more dependent upon electricity and the storage of electrical power.

## 4 Conclusions

The application of biotechnology to the field of mycology and fungal genetics has yielded a great deal of progress in recent years and is on the cusp of applying great positive change to global society. We highlighted the key areas of discovery and application of fungi into emerging technologies. These discoveries and processes have all been made possible by advances in technology that allow for greater understanding and manipulation of fungal genomes and growth conditions. Fungal applications have already shown great promise in terms of their ability to improve efficiency and productivity in various industrial settings. As research continues to progress in this area, it is likely that even more exciting and impactful applications will be discovered. The application of knowledge derived from research on fungi, is poised to be a particularly fruitful arena, with potential applications ranging from the development of new drugs to improvement in agricultural productivity.

## Author contributions

MR, NW, and TB wrote and revised the manuscript. MR and NW generated figures. TB organized and formatted citations and confirmed their accuracy. All authors contributed to the article and approved the submitted version.
